# Early Growth Response 1 Suppresses Macrophage Phagocytosis by Inhibiting NRF2 Activation Through Upregulation of Autophagy During *Pseudomonas aeruginosa* Infection

**DOI:** 10.3389/fcimb.2021.773665

**Published:** 2022-01-12

**Authors:** Zheng Pang, Yan Xu, Qingjun Zhu

**Affiliations:** ^1^ Innovative Institute of Chinese Medicine and Pharmacy, Shandong University of Traditional Chinese Medicine, Jinan, China; ^2^ College of Traditional Chinese Medicine, Shandong University of Traditional Chinese Medicine, Jinan, China; ^3^ Key Laboratory of Traditional Chinese Medicine Classical Theory, Ministry of Education, Shandong University of Traditional Chinese Medicine, Jinan, China

**Keywords:** early growth response 1, *Pseudomonas aeruginosa*, macrophage, phagocytosis, autophagy

## Abstract

*Pseudomonas aeruginosa* is an opportunistic pathogen that causes life-threatening infections in cystic fibrosis patients and immunocompromised individuals. A tightly regulated immune response possessed by healthy individuals can effectively control *P. aeruginosa* infections, whereas the patients with dysregulated immune response are susceptible to this bacterial pathogen. Early growth response 1 (Egr-1) is a zinc-finger transcription factor involved in regulation of various cellular functions, including immune responses. We previously identified that Egr-1 was deleterious to host in a mouse model of acute *P. aeruginosa* pneumonia by promoting systemic inflammation and impairing bacterial clearance in lung, which associated with reduced phagocytosis and bactericidal ability of leucocytes, including macrophages and neutrophils. However, the molecular mechanisms underlying the Egr-1-suppressed phagocytosis of *P. aeruginosa* are incompletely understood. Herein, we investigated whether the Egr-1-regulated autophagy play a role in macrophage phagocytosis during *P. aeruginosa* infection by overexpression or knockdown of Egr-1. We found that overexpression of Egr-1 inhibited the phagocytic activity of macrophages, and the autophagy activator rapamycin and inhibitor chloroquine could reverse the effects of Egr-1 knockdown and Egr-1 overexpression on phagocytosis of *P. aeruginosa*, respectively. Furthermore, the Egr-1-overexpressing macrophages displayed upregulated expression of autophagy-related proteins LC3A, LC3B and Atg5, and decreased levels of p62 in macrophages. Further studies revealed that the macrophages with Egr-1 knockdown displayed enhanced activation of transcription factor NRF2 and expression of scavenger receptors MACRO and MSR1. Altogether, these findings suggest that Egr-1 suppresses the phagocytosis of *P. aeruginosa* by macrophages through upregulation of autophagy and inhibition of NRF2 signaling.

## Introduction

*Pseudomonas aeruginosa* is an environmental ubiquitous bacterium that is harmless to healthy individuals but causes life-threatening acute infections in immunocompromised individuals and chronic infections in cystic fibrosis (CF) patients (Moradali et al., 2017). A tightly regulated innate immune responses possessed by healthy individuals can effectively clear *P. aeruginosa* infections, whereas dysregulated immune responses increase host susceptibility to the infections and cause severe tissue damage (Sadikot et al., 2005). In particular, *P. aeruginosa* induces delayed and persistent inflammation in some disease states, such as chronic obstructive pulmonary disease and CF, leading to chronic bacterial colonization associated with a decline in pulmonary function (Garcia-Clemente et al., 2020). The molecular mechanisms governing the immune responses to *P. aeruginosa* infections remain incompletely understood.

Early growth response 1 (Egr-1), also known as NGFI-A, Krox24, Tis8, Zif268, and ZENK, is a zinc-finger transcription factor that is rapidly and transiently induced by a broad range of extracellular stimuli, including bacterial infections, growth factors, cytokines, stresses, and injury (Dieckgraefe and Weems, 1999; Jones and Agani, 2003; Hoffmann et al., 2008; de Klerk et al., 2017). After activation, Egr-1 translocates to nucleus and binds to a GC-rich consensus sequence, GCG(G/T)GGGCG, in the promotor of target genes, important for immune responses, cell growth, migration, differentiation and apoptosis (Dinkel et al., 1998; Abdel-Malak et al., 2009; Boone et al., 2011; Ten Hoeve et al., 2019). Egr-1 is widely expressed in many different types of cells, and it can function either as a transcriptional activator or a repressor, which depends on cellular context and physiological status (Tan et al., 2003; Qin et al., 2016). As a transcriptional activator, Egr-1 is able to directly mediate target gene transcription by itself or cooperate with other transcription factors such as NF-κB and NFAT through physical interactions (Decker et al., 2003; Ma et al., 2009; Pang et al., 2019). By contrast, binding of transcriptional corepressors NGFI-A binding protein 1 (NAB1) and NAB2 to the inhibitory domain of Egr-1 can repress Egr-1 transcriptional activity (Thiel and Cibelli, 2002).

We previously identified that Egr-1 was deleterious to host in a mouse model of acute lung infection with *P. aeruginosa* by promoting systemic inflammation and impairing bacterial clearance in lung, and Egr-1 deficiency enhanced the phagocytic activity and bactericidal ability of leukocytes, including neutrophils and macrophages (Pang et al., 2019). Similarly, a study by Wu et al. reported that beta-defensin 2 and 3 promoted the phagocytosis of *P. aeruginosa* by macrophages through downregulation of Egr-1 (Wu et al., 2018). Phagocytic clearance is one of the key factors for controlling *P. aeruginosa* infections (Lovewell et al., 2014). Thus, the increased phagocytosis caused by Egr-1 deficiency could be an important factor contributing to clearance of *P. aeruginosa*. Previous studies have shown that autophagy, a conserved degradation process, was able to inhibit the phagocytic activity of macrophages in the context of bacterial infections (Bonilla et al., 2013; O’Keeffe et al., 2015; Zhu et al., 2018). Moreover, Egr-1 has been reported to promote autophagy (Chen et al., 2008; Peng et al., 2016). Therefore, we hypothesize that Egr-1 suppresses macrophage phagocytosis through regulation of *P. aeruginosa*-induced autophagy. In this study, we constructed the macrophage cell lines with Egr-1 overexpression or knockdown, and found that the Egr-1-overexpressing macrophages displayed reduced phagocytic activity. Furthermore, we demonstrated that overexpression of Egr-1 led to increased expression of autophagy-related proteins LC3A, LC3B and Atg5, and decreased levels of p62 in macrophages in response to *P. aeruginosa* infection, suggesting Egr-1 was able to positively regulate the *P. aeruginosa*-induced autophagy. Interestingly, pretreatment of autophagy activator rapamycin (RAP) and autophagy inhibitor chloroquine (CQ) in macrophages could reverse the effect of Egr-1 knockdown and Egr-1 overexpression on phagocytosis of *P. aeruginosa*, respectively. Further study revealed that knockdown of Egr-1 enhanced NRF2 activation, associated with upregulated expression of scavenger receptors MACRO and MSR1. These findings suggest that Egr-1 upregulates *P. aeruginosa*-induced autophagy and decreases accumulation of p62 in macrophages, leading to reduced NRF2 activation associated with downregulated expression of MACRO and MSR1, thus suppressing the phagocytosis of *P. aeruginosa* by macrophages.

## Materials and Methods

### Antibodies

Antibodies for Egr-1 (4154), LC3A (4599), LC3B (83506), Atg3 (3415), Atg4B (5299), Atg5 (12994), Atg7 (8558), Atg12 (4180), Atg16L1 (8089), and NRF2 (12721) were purchased from Cell Signaling Technology. Antibodies for p62 (AF5312), β-Actin (AF0003) and lamin B1(AF1408) were purchased from Beyotime Biotechnology (Shanghai, China).

### Bacterial Preparation

*Pseudomonas aeruginosa* PAO1, a common laboratory strain, was obtained from Wuhan Miaoling Bioscience & Technology (Wuhan, China). *Pseudomonas aeruginosa* was cultured as described previously (Power et al., 2007). Briefly, the suspension cultures were grown in Luria-Bertani (LB) broth overnight at 37°C and shaken at 225 rpm in a shaking incubator (Longyue LYZ-2102, Shanghai, China) until the early stationary phase (optical density [OD] value at 600 nm of between 2.5 to 3). Subsequently, bacteria were washed and resuspended in phosphate-buffered saline (PBS) for experiments.

### Construction of Lentiviral Vector and Transfection

The lentiviral vector for Egr-1 overexpression was constructed based on the full-length protein coding sequence of mouse Egr-1 (GenBank accession no. NM_007913). The Egr-1 sequence was cloned into GV492 vector (GeneChem, Shanghai, China) containing Ubi-MCS-3FLAG-CBh-gcGFP-IRES-puromycin, and the empty GV248 vector (NC1) were used as the negative control. The knockdown lentiviral vector was constructed to express the small interfering RNA (siRNA) for targeting the mouse Egr-1 coding sequence, 5’-GATGGTGGAGACGAGTTAT-3’, and the non-targeting sequence 5’-TTCTCCGAACGTGTCACGT-3’ was used as a negative control (NC2). The Egr-1 siRNA sequence was cloned into GV493 vector containing hU6-MCS-CBh-gcGFP-IRES-puromycin. The lentiviral Egr-1 overexpression or knockdown vector and packaging plasmids (pHelper 1.0 and pHelper 2.0) were cotransfected into 293T cells. The virus-containing supernatants were harvested after 48-72 h transfection, centrifuged at 4000 x g for 10 min at 4°C, and filtered with 0.45 μm pore size filters (EMD Millipore Corp.). Subsequently, mouse macrophage RAW264.7 cells were transduced with the lentiviruses at a multiplicity of infection (MOI) of 30 in complete DMEM (Dulbecco’s modified Eagle medium) supplemented with 10% FBS and 1% Penicillin-Streptomycin (Pen/Strep) for 24 h. Puromycin was added to the cell culture media at a final concentration of 4 ug/ml to eliminate untransduced cells at 72 h post-transduction, and the cells were cultured in presence of this drug for two weeks to obtain stable cell clones. The transduction efficiency was assessed by real-time quantitative PCR (RT-qPCR) and Western blot analysis.

### Cell Culture and *P. aeruginosa* Infection

The stably transduced RAW264.7 cells were maintained in DMEM supplemented with 10% FBS, 1% Pen/Strep and 2 ug/ml puromycin, and media were replaced with antibiotic-free DMEM containing 10% FBS before infection. Cells were infected with *P. aeruginosa* at an MOI of 10 or were mock infected. At different postinfection time points, cell pellets were processed for measurement of the levels of mRNAs or proteins by RT-qPCR or Western blot analysis, respectively. A portion of the cell pellets was reserved for NRF2 activation analysis by ELISA-based transcription activity assay.

### Western Blotting

Cells were lysed in radioimmunoprecipitation assay (RIPA) buffer (Beyotime Biotechnology, P0013B) supplemented with a mixture of protease and phosphatase inhibitors (Beyotime Biotechnology, P1050). Cleared lysates (30 μg protein) were electrophoresed in 10% SDS polyacrylamide gels. Gels were transferred to polyvinylidene difluoride membrane (EMD Millipore Corp., IPVH00010), blocked with 5% nonfat milk powder, probed with primary and secondary antibodies, and detected by an ECL-detection system (GE Healthcare BioSciences Corp.). Blots were quantified using ImageJ software.

### Real-Time Quantitative PCR

Total RNA from cells was purified using an RNeasy kit (Beyotime Biotechnology, R0026), and reverse transcribed into cDNA using a cDNA synthesis kit (Beyotime Biotechnology, D7170M). The *Egr-1* primer sequences were as follows: forward, 5’-CCGCTTTTCTCGCTCGGATG-3’; reverse, 5’-GCGGATGTGGGTGGTAAGGT-3’. The *LC3A* primer sequences were as follows: forward, 5’-CGTCACCCAGGCGAGTTACC-3’; reverse, 5’- AGAGATGCGTCTGCGGTTCG-3’. The *LC3B* primer sequences were as follows: forward, 5’- GTGATCGTCGCCGGAGTCAG-3’; reverse, 5’-CGCTCTATAATCACTGGGATCTTGG-3’. The *NRF2* primer sequences were as follows: forward, 5’-GTCGCCGCCCAGAACTGTAG-3’; reverse, 5’-AAGGTGCTGAGCCGCCTTTT-3’. The *MACRO* primer sequences were as follows: forward, 5’-TGGGCACCCAAAACACACCT-3’; reverse, 5’-TGGACCTGGAGAGCCTCGTT-3’; The *MSR1* primer sequences were as follows: forward, 5’-CTGGGCAGAGCACCCTACTATC-3’; reverse, 5’-AAAAGGTGCCAGGGGATGGGA-3’. The primers were designed by Primer-BLAST (NCBI). RT-qPCR assays were conducted in triplicate using the SYBR green method on QuantStudio 5 Real-Time PCR System (Applied Biosystems) according to manufacturer’s instructions. β-actin was used as housekeeping control mRNA. Data were analyzed using the relative standard curve method according to the manufacturer’s protocol.

### Phagocytosis and Intracellular Killing Assay

Phagocytosis and intracellular killing assays were described previously (Pang et al., 2019). Macrophages were infected with *P. aeruginosa* PAO1 at an MOI of 20. For the phagocytosis assay, cells were collected to enumerate the internalized bacteria after 1 h of incubation. For intracellular killing assay, 100 μg/ml gentamicin was added to the cell culture medium after 1 h postinfection to eliminate the extracellular bacteria. Cells were incubated for another 2 h to evaluate the intracellular killing of bacteria. For both the phagocytosis and intracellular killing assays, cells were washed with PBS, pelleted, and lysed with 0.1% Triton X-100. Cell lysates were serially diluted and plated on LB agar plates for CFU counting.

### Measurement of NRF2 Activation by ELISA

NRF2 activity in cell nuclear extracts was determined using transcription factor ELISA (Mouse NRF2 Transcription Factor Activity Assay Kit, RayBiotech, GA), according to the manufacturer’s instruction. Briefly, nuclear extracts were added into a 96-well plate pre-coated with oligonucleotides containing the NRF2 consensus binding sites, followed by sequential incubations with NRF2 antibody and HRP-labeled secondary antibody. Results were read on a spectrophotometer at 450 nm.

### Statistical Analysis

Data are presented as means ± standard errors of the means (SEM) of the indicated number of experiments. Two-tailed unpaired Student’s t -test was used to compare two independent groups. The statistical significance of comparisons between multiple treatments was determined by one-way analysis of variance and *post hoc* Tukey’s honest significance test. Alternatively, for analysis of two independent variables, a two-way analysis of variance and a Bonferroni multiple-comparison test were used. Statistical analysis was performed using GraphPad Prism software version 5.04 (GraphPad Software Inc., La Jolla, CA).

## Results

### *Pseudomonas aeruginosa* Induced Egr-1 Expression and Autophagy in Macrophages

**Figure 1:**
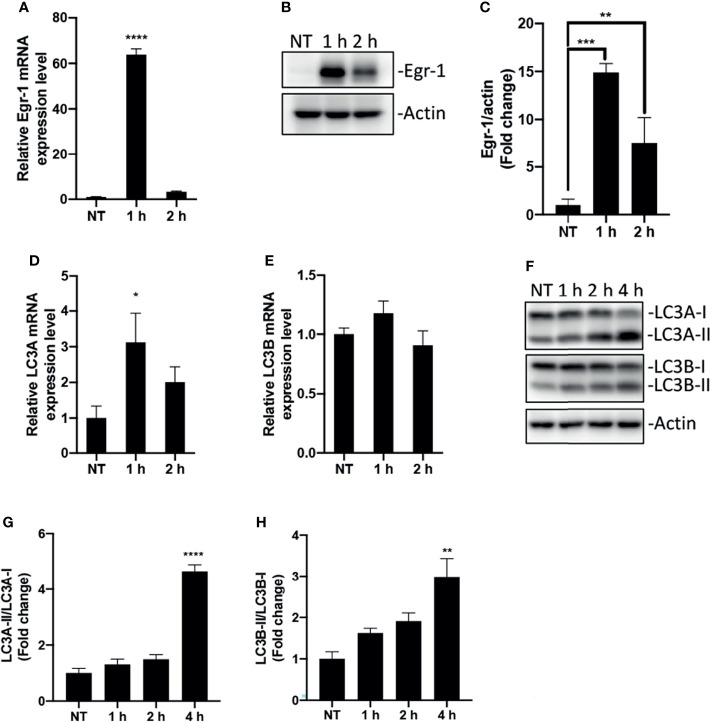
Egr-1 expression and autophagy were induced by *P. aeruginosa* in macrophages. RAW264.7 macrophages were infected with *P. aeruginosa* PAO1 at an MOI of 10 for 1 h, 2 h, 4 h or left untreated (NT). The total RNA isolated from these cells was reverse transcribed to cDNA and subjected to real-time quantitative PCR for assessing the gene expression of Egr-1, LC3A and LC3B. The mRNA levels of Egr-1, LC3A and LC3B were normalized to endogenous control β-actin **(A, D, E)** (n = 3 ± SEM; *p < 0.05; ****p < 0.0001). Cell lysates were subjected to Western blotting for analyzing the protein expression levels of Egr-1, LC3A and LC3B, and actin was used as a loading control. Blots are representative of three independent experiments **(B, F)**. Densitometry analysis of Egr-1 protein levels and the ratio of LC3-II to LC3-I was normalized to actin, and data were presented as fold change **(C, G, H)** (n = 3 ± SEM; *p < 0.05, **p < 0.01, ***p < 0.001, ****p < 0.0001).

Macrophages act as sentinel cells important for host defense against *P. aeruginosa* infections (Cheung et al., 2000; Lovewell et al., 2014). To test whether *P. aeruginosa* induces Egr-1 expression in RAW264.7 macrophages, wild-type RAW264.7 cells were infected with *P. aeruginosa* PAO1 at an MOI of 10 for 1 h, 2 h or were left untreated. We identified that the mRNA and protein expression of Egr-1 in RAW264.7 cells were rapidly and transiently induced by *P. aeruginosa* (**Figures 1A–C**), suggesting Egr-1 may play a role in regulation of *P. aeruginosa*-induced immune responses. Furthermore, Egr-1 is a short-lived protein with a half-life of less than 2 h, and its level is tightly regulated *via* ubiquitin-dependent proteasomal degradation (Bae et al., 2002). Similarly, we observed the rapidly reduced mRNA and protein levels of Egr-1 in RAW264.7 cells after 1 h postinfection (**Figures 1A–C**). Initiation of autophagy is characterized by conversion of cytosolic form of microtubule-associated protein 1A/1B light chain 3 (LC3-I) to a LC3-phosphatidylethanolamine (PE) form (LC3-II), which is correlated with the number of autophagosomes (Mizushima and Yoshimori, 2007). The human LC3 family has three members LC3A, LC3B and LC3C, whereas the mouse LC3 family only contains two members, LC3A and LC3B (Dhingra et al., 2018). Furthermore, the LC3C is poorly characterized and not expressed in most normal tissues, while LC3A and LC3B are differentially expressed in most tissues (Koukourakis et al., 2015). To conform whether *P. aeruginosa* induces autophagy in macrophages, the total RNA and whole cell lysates were extracted from the RAW264.7 cells infected with *P. aeruginosa* PAO1 at an MOI of 10 at various time points as indicated, and subjected to RT-qPCR and Western blot analysis for analyzing the mRNA and protein expression levels of LC3A and LC3B. Results showed that the LC3A and LC3B mRNA expression in macrophages was induced by *P. aeruginosa* (**Figures 1D, E**). Furthermore, we found that the LC3-II/LC3-I ratios for both LC3A and LC3B proteins were significantly increased upon *P. aeruginosa* infection (**Figures 1F–H**), suggesting that *P. aeruginosa* induced autophagy in macrophages.

### Egr-1 Suppressed the Phagocytosis of *P. aeruginosa* by Macrophages

**Figure 2:**
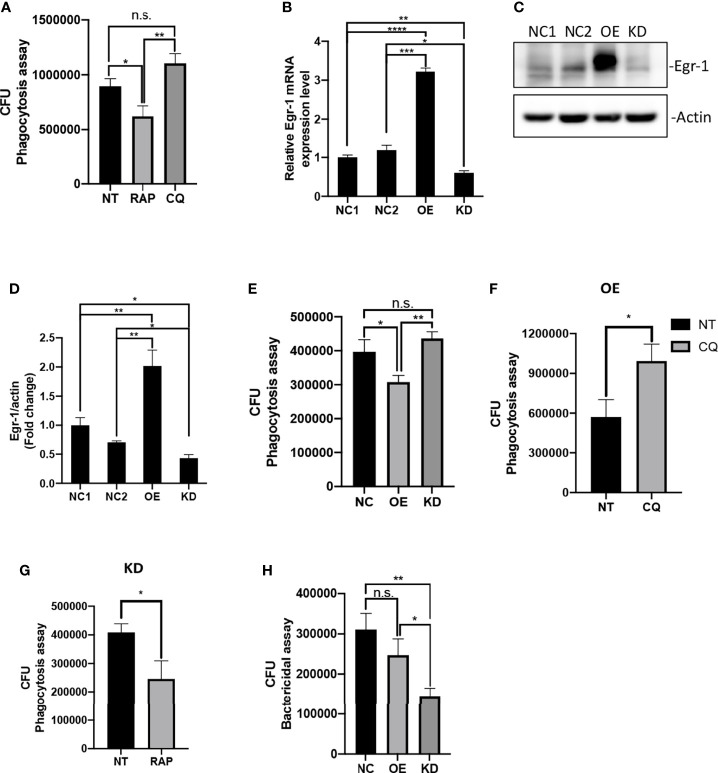
Egr-1 suppressed the phagocytosis of *P. aeruginosa* by macrophages. RAW264.7 macrophages were pretreated with 200 nM rapamycin (RAP) or 20 µM chloroquine (CQ) for 1 h or left untreated (NT). These cells were infected with *P. aeruginosa* PAO1 at an MOI of 20 for 1 h and lysed for phagocytosis assay **(A)**. The CFU data represented the number of internalized bacteria within 1 h (n = 6 ± SEM; n.s., not significant, *p < 0.05, **p < 0.01). The RAW264.7 macrophage cell lines with stable Egr-1 overexpression (OE) or knockdown (KD) were constructed *via* lentiviral transduction. The transduction efficiency was assessed by real-time quantitative PCR and Western blot analysis. The Egr-1 mRNA levels in Egr-1 OE and KD cells as well as their negative controls NC1 and NC2 were normalized to endogenous control β-actin **(B)** (n = 3 ± SEM; *p < 0.05, **p < 0.01, ***p < 0.001, ****p < 0.0001). The blots for Egr-1 protein expression are representative of three independent experiments **(C)**. Densitometry analysis of Egr-1 protein levels was normalized to actin, and data are presented as fold change **(D)** (n=3 ± SEM; *p < 0.05, **p < 0.01). The Egr-1 NC, OE and KD RAW264.7 cells were infected with *P. aeruginosa* PAO1 at an MOI of 20 for 1 h and lysed for phagocytosis assay **(E)** (n = 6 ± SEM; n.s., not significant, *p < 0.05, **p < 0.01). The Egr-1 OE and KD cells were pretreated with 20 μM CQ and 200 nM RAP, respectively, for 1 h or leafed NT. Subsequently, these cells were infected with *P. aeruginosa* PAO1 at an MOI of 20 for 1 h and lysed for phagocytosis assay **(F, G)**. The *P. aeruginosa*-infected Egr-1 NC, OE and KD cells were infected for 3 h and lysed for bacterial killing assay **(H)**. The CFU data represented the number of internalized bacteria survived within the cells after 3 h (n = 6 ± SEM; n.s., not significant, *p < 0.05, **p < 0.01).

Previous studies have shown that increased autophagy could impair phagocytic activity in macrophages (Bonilla et al., 2013; O’Keeffe et al., 2015; Zhu et al., 2018). Similarly, we found that pretreatment of autophagy activator RAP significantly reduced the phagocytic activity of RAW264.7 cells compared to untreated cells, whereas the autophagy inhibitor CQ-pretreated RAW264.7 cells showed a trend of increase in phagocytosis but did not reach statistical significance compared to untreated cells during of *P. aeruginosa* infections (**Figure 2A**). To test whether Egr-1 has an impact on the phagocytosis of *P. aeruginosa* by macrophages, we constructed RAW264.7 cell lines with stable Egr-1 overexpression (OE) or knockdown (KD) *via* lentiviral transduction. RT-qPCR and Western blot analysis were carried out to validate the transduction efficiency. Data showed that both the Egr-1 mRNA and protein levels of Egr-1 OE cells were significantly higher than the mock-transduced cells (NCs) and Egr-1 KD cells, and both the Egr-1 mRNA and protein levels of Egr-1 KD cells were significantly lower than the NC cells as well as Egr-1 OE cells (**Figures 2B–D**). To examine the effects of Egr-1 on the phagocytosis of *P. aeruginosa* by macrophages, the Egr-1 NC, OE and KD RAW264.7 cells were infected with *P. aeruginosa* PAO1 at an MOI of 20 for 1 h. The phagocytic activity of Egr-1 OE cells was significantly decreased compared to Egr-1 NC and KD cells, respectively, and a trend of increase in phagocytic activity of Egr-1 KD cells but did not reach statistical significance compared to NC cells was observed (**Figure 2E**). Interestingly, the phagocytosis patterns of Egr-1 OE and KD were similar to that were generated by autophagy modulators RAP and CQ, respectively, suggesting Egr-1 may influence the *P. aeruginosa*-induced autophagy in macrophages. We further pretreated the Egr-1 OE and KD cells with CQ and RAP, respectively, for 1 h before *P. aeruginosa* infection. The phagocytic activity of CQ-treated Egr-1 OE cells was significantly upregulated compared to untreated Egr-1 OE cells (**Figure 2F**), and the RAP-treated Egr-1 KD cells displayed reduced phagocytic activity compared to untreated Egr-1 KD cells (**Figure 2G**). These data implies that the suppression of macrophage phagocytosis by Egr-1 may be correlated with the increased autophagy. Phagocytosis is a critical process for *P. aeruginosa* clearance (Lovewell et al., 2014). The intracellular killing ability of Egr-1 KD cells was significantly enhanced compared to Egr-1 NC and OE cells (**Figure 2H**).

### Egr-1 Promoted Expression of Autophagy-Related Proteins LC3A, LC3B and Atg5 in Macrophages During *P. aeruginosa* Infection

**Figure 3:**
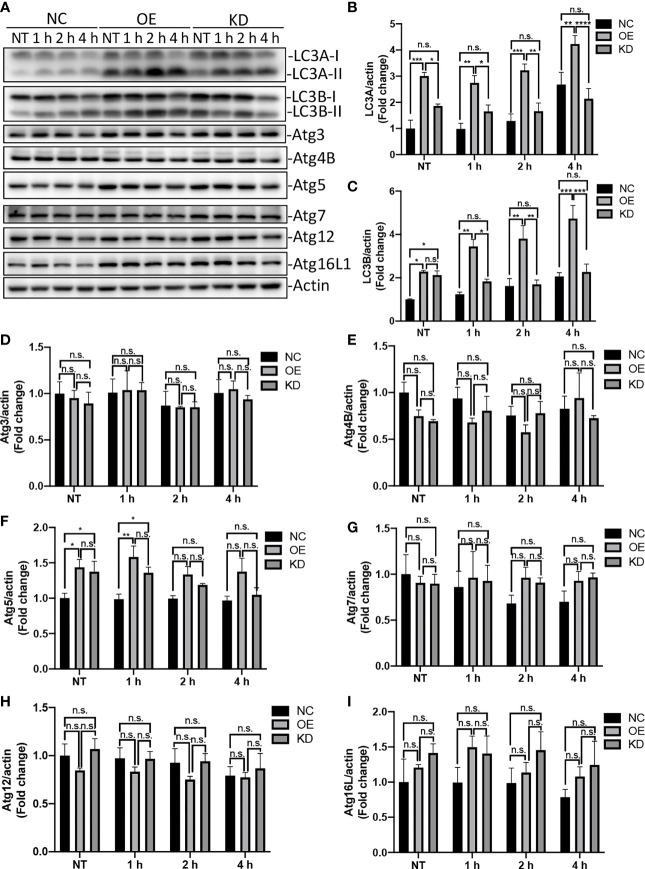
Egr-1 upregulated expression of autophagy-related proteins in macrophages during *P. aeruginosa* infection. Egr-1 NC, OE and KD RAW264.7 cells were infected with *P. aeruginosa* PAO1 at an MOI of 10 for 1 h, 2 h, 4 h or left untreated (NT). Cell lysates were subjected to Western blotting for determining the protein expression levels of LC3A, LC3B, Atg3, Atg4B, Atg5, Atg7, Atg12 and Atg16L1, and actin was used as a loading control. Blots are representative of three independent experiments **(A)**. Densitometry analysis of the protein levels of LC3A **(B)**, LC3B **(C)**, Atg3 **(D)**, Atg4B **(E)**, Atg5 **(F)**, Atg7 **(G)**, Atg12 **(H)** and Atg16L1 **(I)** was normalized to actin, and data were presented as fold change (n = 3 ± SEM; n.s., not significant, *p < 0.05, **p < 0.01, ***p < 0.001, ****p < 0.0001).

Autophagy is an evolutionarily conserved process that degrades and recycles cellular organelles and long-lived proteins in eukaryotic cells (Yin et al., 2016). Atg4B is a cysteine protease that cleave pro-LC3 to generate a C-terminal glycine required for LC3 conjugation to lipids in autophagosomes. Upon induction of autophagy, the cytosolic LC3-I is activated by the E1 enzyme Atg7 and transferred to E2 enzyme Atg3, which catalyzes the covalent conjugation of LC3-I to PE with the assistance of the Atg12-Atg5-Atg16 complex (Tanida et al., 2014; Ye et al., 2018). Thereafter, the PE-conjugated LC3, LC3-II, is recruited to the membrane of nascent autophagosome for cargo recognition (Song et al., 2019). To test whether Egr-1 affects the expression of autophagy-related proteins, the Egr-1 NC, OE, KD RAW264.7 cells were infected with *P. aeruginosa* PAO1 at an MOI of 10 for 1 h, 2 h, 4 h or left untreated, the whole cell lysates were subjected to Western blot analysis for determining the protein levels of LC3A, LC3B, Atg3, Atg4B, Atg5, Atg7, Atg12 and Atg16L1. The results showed that Egr-1 OE cells manifested significantly elevated protein levels of LC3A, LC3B and Atg5 compared to Egr-1 NC cells during *P. aeruginosa* infection (**Figures 3A–C, F**), whereas no significant differences were observed in the protein expression levels of Atg3, Atg4B, Atg7, Atg12 and Atg16L among these cells (**Figures 3A, D, E, G–I**), suggesting that Egr-1 could promote the *P. aeruginosa*-induced autophagy through upregulation of the key autophagy-related proteins LC3A, LC3B and Atg5. Interestingly, the protein expression level of Atg5 at 1 h postinfection in Egr-1 KD cells was higher than that was in Egr-1 NC cells but showed no significant difference compared to Egr-1 OE cells (**Figure 3F**), implying that Atg5 may also be regulated by other transcription factors, which compensate the effect caused by Egr-1 deficiency.

### Egr-1 Suppressed NRF2 Activation Through Reducing the Levels of p62 in Macrophages in Response to *P. aeruginosa* Infection

**Figure 4:**
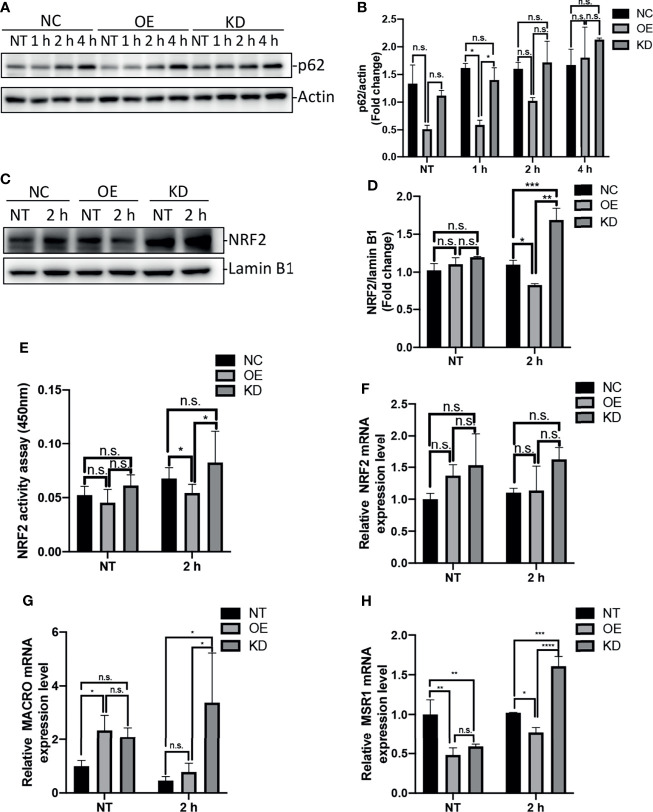
Egr-1 suppressed NRF2 activation through reducing the levels of p62 in macrophages in response to *P. aeruginosa* infection. Egr-1 NC, OE and KD RAW264.7 cells were infected with *P. aeruginosa* PAO1 at an MOI of 10 for 1 h, 2 h, 4 h or left untreated (NT). Cell lysates were subjected to Western blotting for determining the p62 protein expression levels, and actin was used as a loading control. Blots are representative of three independent experiments **(A)**. Densitometry analysis of the p62 protein levels was normalized to actin, and data were presented as fold change **(B)** (n = 3 ± SEM; n.s., not significant, *p < 0.05). The nuclear proteins were extracted from the Egr-1 NC, OE and KD cells infected with *P. aeruginosa* PAO1 for 2 h or left untreated, and subjected to Western blot analysis for examining the protein levels of NRF2 in nucleus. Blots are representative of three independent experiments **(C)**. Densitometry analysis of the NRF2 protein levels in nucleus was normalized to lamin B1, and data were presented as fold change **(D)** (n = 3 ± SEM; n.s., not significant, *p < 0.05, **p < 0.01, ***p < 0.001). The nuclear extracts from NT and *P. aeruginosa* 2 h infected Egr-1 NC, OE and KD cells were subjected to transcription factor ELISA for determining NRF2 activity **(E)** (n = 6 ± SEM n.s., not significant, *p < 0.05). The total RNA isolated from NT and *P. aeruginosa* 2 h infected Egr-1 NC, OE and KD cells were reverse transcribed to cDNA and subjected to real-time quantitative PCR for analyzing the mRNA transcription levels of NRF2, MACRO and MSR1. The mRNA levels of NRF2, MACRO and MSR1 were normalized to endogenous control β-actin **(F–H)** (n = 3 ± SEM; n.s., not significant, *p < 0.05, **p < 0.01, ***p < 0.001, ****p < 0.0001).

The protein p62, also known as SQSTM1, is an autophagy-adaptor protein that is responsible for recruitment of ubiquitinated cargo to autophagosome for degradation (Liu et al., 2016). Moreover, this protein is degraded by autophagy, and accumulates in cytosol when autophagy is inhibited (Bjorkoy et al., 2009). Previous studies have shown that accumulation of p62 was able to induce activation of transcription factor NRF2 and expression of NRF2 target genes (Bonilla et al., 2013; Ichimura et al., 2013). To examine the impact of Egr-1 on p62 protein levels in macrophages, the Egr-1 NC, OE and KD RAW264.7 cells were infected with *P. aeruginosa* PAO1 at an MOI of 10 for 1 h, 2 h, 4 h or left untreated. We found that the Egr-1 OE cells displayed significantly reduced protein levels of p62 at 1 h compared to Egr-1 NC and KD cells, respectively (**Figures 4A, B**). To analyze the NRF2 activation in macrophages, the nuclear extracts from the *P. aeruginosa*-infected Egr-1 NC, OE and KD cells were subjected to Western blot analysis and an ELISA-based NRF2 activity assay. Data showed that the protein level of NRF2 in nucleus at 2 h postinfection in Egr-1 KD cells was significantly higher than those in Egr-1 NC and OE cells. By contrast, Egr-1 OE cells displayed reduced NRF2 protein level in nucleus at 2 h postinfection compared to Egr-1 NC and KD cells, respectively (**Figures 4C, D**). Similarly, the decreased NRF2 DNA-binding activity at 2 h postinfection was observed in Egr-1 OE cells compared to Egr-1 NC and KD cells, respectively (**Figure 4E**). To determine whether Egr-1 regulates NRF2 gene expression, we assessed the mRNA levels of NRF2 in Egr-1 NC, OE and KD cells upon *P. aeruginosa* infection, and no significant differences were observed among the three cell lines (**Figure 4F**). These findings suggest that Egr-1 suppressed NRF2 activation through downregulation of p62 but did not affect NRF2 expression.

NRF2 has been previously reported to regulate macrophage phagocytosis through upregulation of scavenger receptors MACRO and MSR1 (Reddy et al., 2009; Harvey et al., 2011; Bonilla et al., 2013). We further identified that the mRNA levels of MACRO and MSR1 at 2 h postinfection were significantly increased in Egr-1 KD cells compared to Egr-1 NC and OE cells, respectively (**Figures 4G, H**), which was similar to the pattern of NRF2 nuclear protein levels (**Figure 4D**).

## Discussion

**Figure 5:**
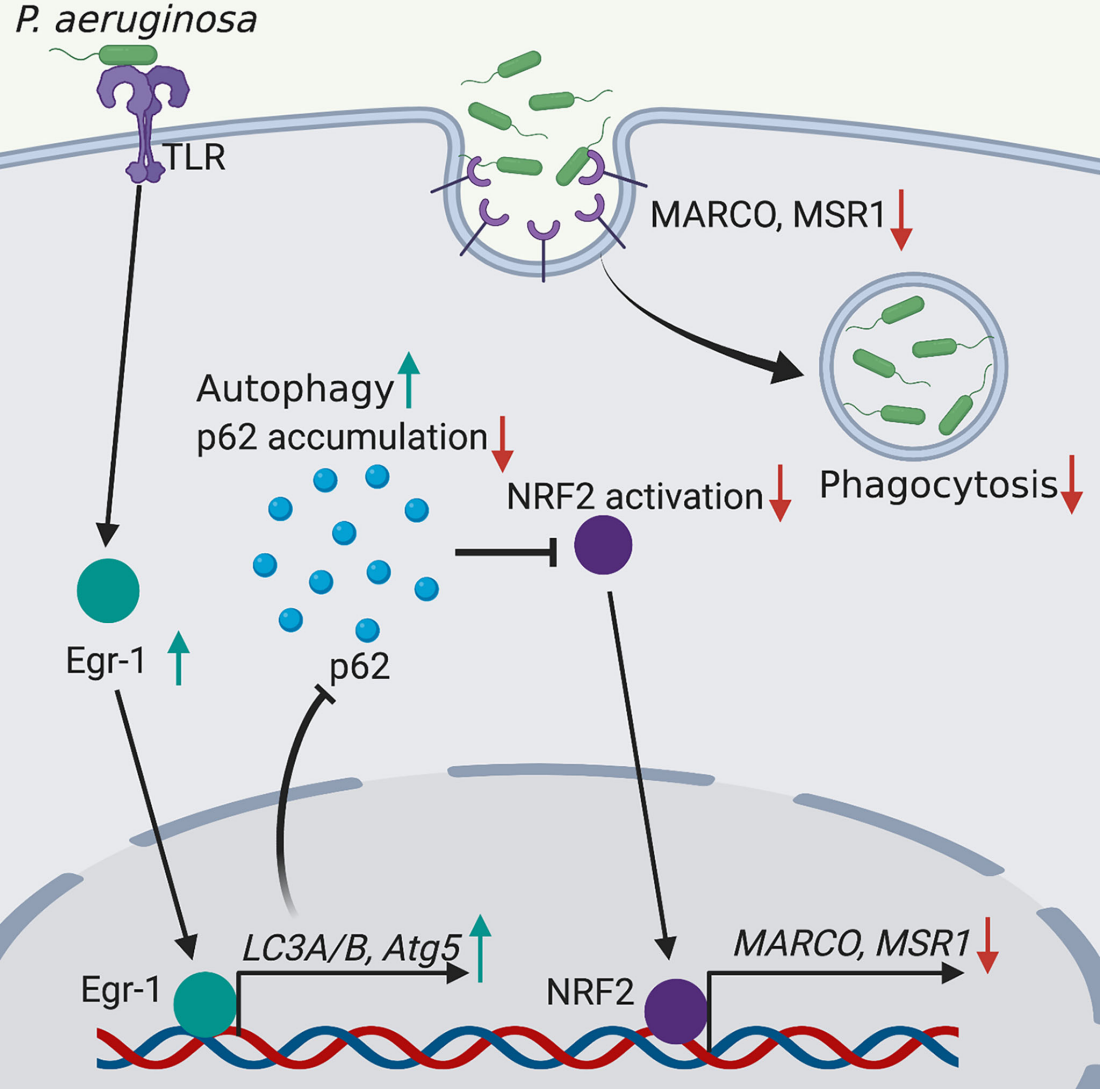
Schematic illustration of Egr-1-regulated macrophage phagocytosis of *P. aeruginosa*. Egr-1 enhances the *P. aeruginosa*-induced autophagy by upregulation of the autophagy-related proteins LC3A, LC3B and Atg5 and decreases accumulation of p62, leading to reduced NRF2 activation associated with downregulated expression of scavenger receptors MACRO and MSR1, thus suppressing the phagocytosis of *P. aeruginosa* by macrophages.

Phagocytosis is a critical process for engulfing and eliminating of pathogens, and maintaining host tissue homeostasis, and this process is accomplished by the professional phagocytes such as monocytes, macrophages, dendritic cells and neutrophils (Uribe-Querol and Rosales, 2020). Deficiency in phagocytosis increases host susceptibility to *P. aeruginosa* infections and causes delayed bacterial clearance (Lovewell et al., 2014). The molecular mechanisms involved in regulation of phagocytosis are incomplete understood. The transcription factor Egr-1 regulates many cellular processes and functions, including immune responses (Dinkel et al., 1998; Abdel-Malak et al., 2009; Boone et al., 2011; Ten Hoeve et al., 2019). Moreover, the activation and expression of Egr-1 have been found to be induced by bacterial adhesion (de Klerk et al., 2017). We and others have previously identified that Egr-1 deficiency could enhance the phagocytosis of *P. aeruginosa* by leucocytes including macrophages and neutrophils (Wu et al., 2018; Pang et al., 2019). In this study, we continued to explore the mechanisms underlying the Egr-1-suppressed phagocytosis of *P. aeruginosa* by overexpression or knockdown of Egr-1 in macrophages, and found that Egr-1 suppressed macrophage phagocytosis of *P. aeruginosa* through upregulation of autophagy. Specifically, the elevated Egr-1 expression enhanced *P. aeruginosa*-induced autophagy by upregulation of LC3A, LC3B and Atg5, leading to reduced p62 level and NRF2 activation, associated with decreased expression of scavenger receptors MACRO and MSR1 (**Figure 5**).

Previous studies have shown that *P. aeruginosa* was able to induce autophagy in many kinds of leukocytes, including neutrophils, macrophages and mast cells (Yuan et al., 2012; Junkins et al., 2013; Itoh et al., 2015). Formation of autophagosome involves approximately 20 core autophagy-related proteins, which collaboratively regulate initiation of autophagy, nucleation of isolation membrane (phagophore), elongation of the isolation membrane and closure to form a double membrane vesicle (Mehrpour et al., 2010). Among these autophagy-related proteins, LC3 is considered as a reliable autophagosomal marker, which primarily exists as a cytosolic form LC3-I and conjugated to PE to form LC3-II upon induction of autophagy (Yin et al., 2016). Subsequently, LC3-II is recruited to the membrane of autophagosome for cargo recognition by binding to cargo receptor p62 (Song et al., 2019). LC3 exists two isoforms LC3A and LC3B in mouse, whereas human has an additional isoform LC3C, which is transcribed at low levels with limited tissue distribution (He et al., 2003). Additionally, these three LC3 isoforms are differentially expressed in normal tissues and responded differently to extracellular stimuli (He et al., 2003; Wang et al., 2013), indicating that the roles of LC3 isoforms vary in different cellular context. Our data showed that both LC3A-I and LC3B-I were converted to their PE-conjugated form in mouse macrophages in response to *P. aeruginosa* infections, suggesting that both LC3A and LC3B were involved in *P. aeruginosa*-induced autophagic process, and served as autophagosomal markers. In mammals, Atg5 forms a complex with Atg12 and Atg16L1, which regulates formation of autophagosome by facilitating the LC3-PE conjugation (Mizushima et al., 2011). Furthermore, the Atg5-Atg12-Atg16 complex is also able to directly tether on the membrane of phagophore independently of LC3, which promotes autophagosome-lysosome fusion through the interaction between Atg5 and a tethering coherent protein TECPR1 on lysosome membrane (Chen et al., 2012; Ye et al., 2018). Importantly, Atg5 deficiency results in inhibition of autophagy, indicating that Atg5 is a key autophagy protein (Ye et al., 2018). In this study, we identified the upregulated protein levels of LC3A, LC3B and Atg5 in Egr-1 OE RAW264.7 cells compared to Egr-1 NC RAW264.7 cells, suggesting Egr-1 mediated *P. aeruginosa*-induced autophagy by enhancing the expression of LC3A, LC3B and Atg5 in macrophages. Interestingly, no significant differences of LC3A and LC3B protein levels were observed between Egr-1 KD and NC cells, and the Egr-1 KD cells displayed increased Atg5 protein expression at 2 h postinfection compared to Egr-1 NC cells. This could be explained by the fact that the transcriptional regulation of autophagy is mediated by multiple transcription factors, including NF-κB, ATF4, FoxOs and MiTF/TFE family (Anderson and Macleod, 2019). The effects of downregulated Egr-1 on LC3A, LC3B and Atg5 expression may be compensated by other transcription factors.

The protein p62 is a cargo receptor that interacts with LC3 for delivery of ubiquitinated protein aggregates to autophagosome for autophagic degradation (Liu et al., 2016). Moreover, p62 is also degraded together with the protein aggregates during autophagy (Puissant et al., 2012). Induction of autophagy reduces the level of p62, while inhibition of autophagy results in accumulation of p62 (Bjorkoy et al., 2009). Thus, p62 can be used as an autophagy marker. Our data revealed that overexpression of Egr-1 significantly reduced the protein levels of p62 in macrophages during *P. aeruginosa* infection, which confirmed the role of Egr-1 in regulation of *P. aeruginosa*-induced autophagy in macrophages. The transcription factor NRF2 is a master regulator of cytoprotective responses to environmental stresses (Hiebert, 2021), and is tightly regulated by Keap-1, which targets NRF2 for ubiquitination and degradation (Jiang et al., 2015). Furthermore, the Keap1-interacting region domain of p62 binds to Keap-1, leading to dissociation of NRF2 from Keap-1, allowing NRF2 nuclear translocation and target gene expression (Jiang et al., 2015; Tonelli et al., 2018). Activation of NRF2 has been implicated in host defense against bacterial infections (Harvey et al., 2011; Kong et al., 2011; Nakajima et al., 2021). Our findings showed that the reduced p62 level associated with decreased NRF2 nuclear protein levels and DNA-binding activity in Egr-1 OE macrophages. Moreover, the Egr-1 KD macrophages exhibited great increased nuclear protein levels of NRF2 compared to Egr-1 NC and OE cells, respectively. Given the ability of Egr-1 modulating the activation of other transcription factors through physical interactions (Chapman and Perkins, 2000; Decker et al., 2003), it is also possible that Egr-1 directly inhibits NRF2 activation and sequesters it from nuclear translocation or binding to the promoters of target genes.

Oxidative stress is able to induce NRF2 expression (Kobayashi et al., 2006), and target Keap1-NRF2 complex, leading to dissociation of NRF2 from Keap-1 in cytosol (Kobayashi and Yamamoto, 2005; Kaspar et al., 2009). Furthermore, *P. aeruginosa* infection induces oxidative stress by producing virulence factors, such as LPS, pyocyanin, ExoU and Pseudomonas quinolone signal (PQS), leading to release of reactive oxygen species (ROS) and reactive nitrogen species (RNS) from phagocytes (Caldwell et al., 2009; da Cunha et al., 2015; Abdalla et al., 2017; Liu et al., 2017). We previously identified that Egr-1 deficiency caused an upregulated levels of nitric oxide in the lung tissues of mice and phagocytes including neutrophils and macrophages upon *P. aeruginosa* infection (Pang et al., 2019). It is possible that the elevated oxidative stress caused by Egr-1 deficiency contributed to the increased NRF2 activation. In addition to acute infectious diseases, NRF2 also plays a critical role against a variety of chronic diseases including cardiovascular diseases, chronic liver diseases, chronic obstructive pulmonary disease and chronic kidney diseases by reducing oxidative stress *via* regulation of the cytoprotective genes encoding detoxifying enzymes and antioxidants (Kaspar et al., 2009; Small et al., 2012; Ezhilarasan, 2018; Sharifi-Rad et al., 2020; Wiegman et al., 2020).

MACRO and MSR1 are class A scavenger receptors that are predominantly expressed in macrophages and important for phagocytosis and clearance of *P. aeruginosa* (Reddy et al., 2009; Harvey et al., 2011). Moreover, previous studies have shown that NRF2 played a critical role in regulation of bacterial phagocytosis by upregulation of the scavenger receptors MACRO and MSR1 in macrophages (Reddy et al., 2009; Harvey et al., 2011; Bonilla et al., 2013; Liu et al., 2019). In consistent to the pattern of NRF2 protein levels in nucleus, the mRNA transcription levels of MACRO and MSR1 were significantly elevated in *P. aeruginosa*-infected Egr-1 KD macrophages compared to Egr-1 NC macrophages, suggesting that the upregulated NRF2 activation caused by Egr-1 knockdown could enhance the expression of MACRO and MSR1 in macrophages, thus leading to increased phagocytosis of *P. aeruginosa*.

Altogether, our findings demonstrated a novel regulatory mechanism of Egr-1-mediated macrophage phagocytosis during *P. aeruginosa* infection, which enhanced the *P. aeruginosa*-induced autophagy by upregulation of the autophagy key proteins LC3A, LC3B and Atg5, leading to decreased activation of NRF2 and reduced expression of MACRO and MSR1. This study broadens our understanding of the molecular mechanisms involved in regulation of phagocytosis in innate immunity, and suggests that inhibition of Egr-1 could be a potential therapeutic approach for treatment of *P. aeruginosa* infections, which enhances bacterial clearance.

## Data Availability Statement

The original contributions presented in the study are included in the article/supplementary material. Further inquiries can be directed to the corresponding author.

## Author Contributions

ZP contributed to the project administration, experimental design and performance, data analysis and manuscript writing. YX performed experiments. QZ contributed to manuscript writing. All authors contributed to the article and approved the submitted version.

## Funding

This study was funded by the National Natural Science Foundation of China (Grant No. 82002112).

## Conflict of Interest

The authors declare that the research was conducted in the absence of any commercial or financial relationships that could be construed as a potential conflict of interest.

## Publisher’s Note

All claims expressed in this article are solely those of the authors and do not necessarily represent those of their affiliated organizations, or those of the publisher, the editors and the reviewers. Any product that may be evaluated in this article, or claim that may be made by its manufacturer, is not guaranteed or endorsed by the publisher.

## Abbreviations

CF, cystic fibrosis; NAB1, NGFI-A binding protein 1; RAP, rapamycin; CQ, chloroquine; OD, optical density; siRNA, interfering RNA; MOI, multiplicity of infection; DMEM, Dulbecco’s modified Eagle medium; Pen/Strep, Penicillin-Streptomycin; RIPA, radioimmunoprecipitation assay; RT-qPCR, real-time quantitative PCR; PBS, phosphate-buffered saline; SEM, standard errors of the means; LC3, microtubule-associated protein 1A/1B light chain 3; PE, phosphatidylethanolamine; OE, overexpression; KD, knockdown; NC, negative control; PQS, Pseudomonas quinolone signal; ROS, reactive oxygen species; RNS, reactive nitrogen species.
